# Dieting is associated with reduced bone mineral accrual in a longitudinal cohort of girls

**DOI:** 10.1186/s12889-018-6206-y

**Published:** 2018-11-22

**Authors:** Emily E. Hohman, Katherine N. Balantekin, Leann L. Birch, Jennifer S. Savage

**Affiliations:** 1 Center for Childhood Obesity Research, The Pennsylvania State University, 129 Noll Laboratory, University Park, PA 16802 USA; 2 Department of Exercise and Nutrition Sciences, University at Buffalo, 12 Sherman Hall, Buffalo, NY 14214 USA; 3 Department of Foods and Nutrition, The University of Georgia, 172 Dawson Hall, Athens, GA 30602 USA; 4 Center for Childhood Obesity Research and Department of Nutritional Sciences, The Pennsylvania State University, 103 Noll Laboratory, University Park, PA 16802 USA

**Keywords:** Eating behavior, Dieting, Children, Adolescence, Bone

## Abstract

**Background:**

Peak bone mass accrual occurs during adolescence, a time when dieting and related eating behaviors are common. Impaired bone mineral accrual is a known consequence of eating disorders in adolescents, but the effects of subclinical dieting behaviors on bone mineral content (BMC) have not been described in this age group. The goal of this analysis was to determine whether dieting behavior in preadolescence and adolescence is associated with bone mineral accrual in adolescent girls.

**Methods:**

Non-Hispanic white girls (*n* = 139) were followed in a longitudinal cohort study. BMC was assessed at ages 9 and 15y. Dieting to lose weight was reported every 2 years, and dietary restraint and disinhibition, eating attitudes, weight concerns, and body esteem were assessed at age 11y. Girls were classified as “early dieters” if they first dieted by age 11y (31.7%), “adolescent dieters” if they first dieted after 11y (46.8%), or non-dieters if they did not report dieting by 15 y (21.6%). The effect of dieting related variables on BMC at 15y and change in BMC from 9 to 15y was assessed using linear regression, controlling for height, weight, BMI, physical activity, and pubertal status.

**Results:**

Girls who first reported dieting to lose weight by age 11y had a 4.2% lower bone mineral accrual across adolescence (*p* = 0.02) and 3.1% lower BMC at age 15y (*p* = 0.005) than girls who first reported dieting after 11y or not at all. Number of weight control behaviors used, dietary restraint, and weight concerns were also negatively associated with BMC (*p* < 0.05).

**Conclusions:**

Dieting behavior in preadolescence is associated with reduced bone mineral accrual. Strategies to promote optimal bone development should include prevention of dieting.

**Trial registration:**

Clinicaltrials.gov NCT03342430, November 17, 2017. Retrospectively registered.

**Electronic supplementary material:**

The online version of this article (10.1186/s12889-018-6206-y) contains supplementary material, which is available to authorized users.

## Background

Adolescence is a critical period for bone development, with nearly 40% of adult skeletal calcium accumulated in the 4 years surrounding peak height velocity [1]. In healthy white girls, peak calcium accretion occurs at a mean of 12.5 years [2]. Maximizing bone mineral accrual during this time may result in greater adult bone mineral density (BMD) and protection against osteoporosis and fracture later in life. Non-modifiable factors such as sex, race/ethnicity, and genetics account for 60–80% of variance in peak bone mass, while environmental and lifestyle factors contribute the remaining 20–40% [3]. Diet and physical activity have been the most extensively studied behavioral contributors to peak bone mass accrual, but relationships between bone accrual and other bio-behavioral factors have not been well characterized [3].

Weight concerns, dietary restraint, and self-initiated dieting/weight control behaviors also emerge during preadolescent and adolescent years [4]. Young girls are aware of dieting behaviors [5], and concerns about weight and body dissatisfaction emerge as early as age 5 [6] and predict later dietary restraint and dieting attempts in preadolescent girls [7]. Over 60% of adolescent girls report trying to lose weight [8]. Dieting in adolescence has been associated with increased risk of both overweight and eating disorders [9]. In adults, dieting has been associated with reductions in bone density [10, 11]. Low bone density is a well-documented consequence of eating disorders in adolescents [12], but effects of subclinical weight control behaviors on bone development have not been well studied in this age group. The objectives of this analysis were to determine if gain in bone mineral content (BMC) from 9 to 15 years and BMC at 15 years are related to self-reported dieting, eating, and weight-related behaviors (e.g. restraint, disinhibition) in a longitudinal sample of girls. We hypothesized that girls who reported dieting would have smaller gains in BMC from 9 to 15 years and lower BMC at 15 years than girls who did not report dieting.

## Methods

### Participants

Participants were part of a longitudinal cohort study of the health and development of young girls, consisting of 197 non-Hispanic white 5-year-old girls living in central Pennsylvania. Families were recruited for participation in the study in 1996–97 through flyers and newspaper advertisements. Additionally, families with age-eligible daughters within a five-county radius received mailings and follow-up phone calls. Eligibility criteria for girls’ participation at the time of recruitment included living with both biological parents, absence of severe food allergies or chronic medical conditions affecting food intake, and the absence of dietary restrictions involving animal products. Girls were assessed every 2 years from age 5 to age 15 years, though bone mineral content was first assessed at age 9. At age 15, *n* = 167 girls remained in the study, and attrition was primarily due to relocation outside the area. For this analysis, girls who had bone mineral content data at ages 9 and 15 as well as complete anthropometric, pubertal status, and physical activity data were included, resulting in a sample of 139 girls. This sample size yields 80% power to detect a medium size effect of dieting status based on Cohen’s f (0.24). [13] Compared to participants who dropped before age 15 or were excluded from this analysis due to missing data (*n* = 58), those that were included had slightly older mothers (mean (SD) 35.8 (4.7) vs. 34.2 (4.8) years at enrollment, *p* = 0.03), but there were no differences in parent income, education, and BMI, paternal age, or girl’s BMI percentile at enrollment. Additional details on this cohort have been published elsewhere [5, 14, 15]. The Pennsylvania State University Institutional Review Board approved all procedures, and parents provided written consent for their daughters’ participation.

### Physical measurements

Height and weight were measured in triplicate by trained staff using calibrated scales at each assessment and were used to calculate BMI. BMI percentiles were calculated using the 2000 CDC growth charts [16]. Pubertal status via breast development was assessed by a nurse using the Tanner rating system [17]. Total body BMC was assessed using dual energy X-ray absorptiometry (DXA). A trained technician obtained measurements with participants in a supine position in light clothing without shoes. Whole-body scans were obtained using a Hologic QDR 4500 W instrument in the array mode, and scans were analyzed using QDR4500 Whole Body Analysis Software. Coefficients of variation for this instrument were determined to range from 0.7% for total hip BMD to 1.4% for femoral neck BMD, which is consistent with other reports of precision for this model [18, 19]. BMC rather than BMD was used as an outcome measure based on previous reports that BMC is a more accurate and reliable measure of bone acquisition in children [20].

### Physical activity

Habitual physical activity was assessed at age 9 using a checklist of 22 different activities [21]. Girls were asked to indicate whether they participated in various sport and leisure activities (e.g. basketball, swimming, gymnastics, roller-blading), and if so, how many times per week. The total frequency of activities per week was calculated.

### Dietary assessment

Dietary intake of calcium and other nutrients was assessed using 24-h dietary recalls completed at each biennial assessment [15]. Three 24-h recalls were obtained at each time point, on 2 weekdays and 1 weekend day selected randomly over a 2-week period. Interviews were conducted by telephone by trained staff at the Pennsylvania State University Diet Assessment Center using a computer-assisted, multiple pass approach (Nutrition Data System for Research (NDS-R), Nutrition Coordinating Center, University of Minnesota, Minneapolis, MN). Both mothers and daughters were present for interviews; at age 9, the mother served as the primary reporter with input from the daughter, and at ages 11, 13, and 15, the daughter was the primary reporter with input from the mother as needed. Average nutrient intakes were calculated using NDS-R. In the present study, we examined nutrients that have been associated with bone health in previous studies in children and adolescents [3].

### Dieting behaviors

Self-reported dieting to lose weight was assessed using a dichotomous yes/no question, “Have you ever dieted?”, at each visit. Girls were instructed that they should think of a diet as “whenever you eat less or exercise more in order to lose weight.” Girls who first reported dieting by age 11 were classified as “early dieters,” and girls who first reported dieting between 11 and 15 years were classified as “adolescent dieters.” Girls who did not report dieting by age 15 were classified as “non-dieters” [14]. Girls were also asked if they had ever engaged in a variety of healthy (e.g., increase exercise, increase fruit and vegetables) and unhealthy (e.g., skip meals, laxatives) weight control behaviors. The list of weight control behaviors was adapted from the comprehensive list developed by French et al. [22]. The number of weight control behaviors used was totaled, with a greater number of behaviors used indicating greater intensity of weight control behavior. Previous work in this cohort found that increasingly intense patterns of dieting, including those characterized by primarily “healthy” strategies, were associated with poorer psychological outcomes (e.g., depressive symptoms, self-esteem) [23].

Dietary restraint and disinhibition were measured using the Dutch Eating Behavior Questionnaire (DEBQ) [24]. Disordered eating behaviors were measured using the Children’s Eating Attitude Test (chEAT). Total scores as well as subscale scores for Dieting, Food Preoccupation, and Social Pressure to Eat were calculated [25]. Weight concerns were assessed using the Weight Concerns Scale [26]. Weight-related body esteem was assessed using the Body-Esteem Scale for Adolescents and Adults [27]. Though these constructs were assessed at multiple ages in this longitudinal cohort, measurements at age 11 years were used to correspond with the measure of early-onset dieting, and because they represented behaviors occurring prior to the timing of peak bone mineral accrual.

### Statistical analysis

Analyses were performed in SAS 9.4. Descriptive statistics were generated for all variables of interest. Effects of dieting, behavioral measures, and dietary intake on change in BMC from 9 to 15 years (ΔBMC) and absolute BMC at 15 years were assessed using a general linear model (PROC GLM). Established predictors were first entered as a block into the model. When ΔBMC from age 9 to 15 years was used as the outcome, predictors included BMC at 9 years, change in height from age 9 to 15 years, change in weight from age 9 to 15 years, breast Tanner score at age 9 years, BMI percentile at age 15 years, and frequency of participation in physical activity at 9 years. For BMC at 15 years, the predictors were BMC at age 9, height at age 15, breast Tanner score at age 9, BMI percentile at age 15 years (weight at 15 did not predict BMC at 15 independently of BMI at 15), and frequency of physical activity at 9 years. Next, dieting variables were then added to the regression individually. Variables that were statistically significant in the individual models were then examined together. A final model for each outcome was selected using a stepwise selection process (PROC GLMSELECT). Assumptions of linearity and homoscedasticity were confirmed by examinations of residuals. To further examine potential contributors to the effect of early dieting on bone mineral accrual, differences in weight control behaviors and nutrient intake between early, adolescent, and non-dieters were assessed using Fisher’s exact test or ANOVA and Tukey’s post-hoc test. All tests were parametric and two-tailed. Significance was accepted at *p* < 0.05.

## Results

### Participant characteristics

Participant characteristics are described in Table 1. On average, girls gained 1104 g (SD 168.3) of bone mineral from age 9 to 15 years. Most girls had a BMI percentile in the normal weight range (5th- < 85th). The percentage of girls with overweight or obesity (≥85th percentile) declined slightly from 28.8% (*n* = 40) at age 9 to 21.6% (*n* = 30) at age 15. The majority of girls reported dieting at some point during the study, with 31.7% (*n* = 44) of girls reporting dieting by age 11 (‘early dieters’), and 46.8% (*n* = 65) of girls first reporting dieting between 11 and 15 (‘adolescent dieters’). Only 21.6% (n = 30) did not reported dieting at any time point.

**Table 1:** Participant characteristics at 9 and 15 years of age (*n* = 139)

	9 years	15 years	Change from 9 to 15 years
Mean (SD)			
Height (cm)	136.2 (6.2)	164.6 (6.6)	28.4 (4.3)
Weight (kg)	34.3 (7.4)	60.2 (11.9)	25.9 (8.8)
BMI percentile	64.2 (26.6)	61.1 (25.0)	−3.1 (21.2)
Total body BMC (g)	952 (147)	2055 (260)	1104 (168)
BMD (g/cm^2^)	0.837 (0.045)	1.090 (0.075)	0.253 (0.049)
Breast Tanner score	1.76 (0.8)	NA	
Physical activity (times participated/week)	12.8 (7.7)	NA	
N (%)			
% overweight (≥85th percentile)	40 (28.8)	30 (21.6)	
Family income^1^			
<$35,000	22 (15.9)	15 (11.0)	
$35,000–$50,000	40 (29.0)	21 (15.3)	
>$50,000	76 (55.1)	101 (73.7)	
Parents married	129 (92.8)	122 (88.4)	

Values are mean (SD) or N (%). NA = not assessed

^1^Age 9 data collected in 2000–2001, age 15 data collected in 2006–2007

Differences in weight control behaviors at age 11y by dieting onset category are presented in Table 2**.** Girls who were classified as early dieters engaged in more weight control strategies, and had higher restraint, disinhibition, chEAT dieting and total scores, and weight concerns, and lower weight-related body esteem, than girls classified as adolescent dieters or non-dieters. Though both the adolescent dieters and non-dieter groups had not dieted at age 11, there were some characteristics that distinguished these groups at this age. Adolescent dieters had significantly higher restraint and weight concerns at age 11 years than non-dieters, suggesting elevated levels of these characteristics at 11 years preceded onset of dieting between 11 and 15 years.

**Table 2:** Eating and weight control behaviors at age 11y by onset of dieting category

Variable	Early dieters	Adolescent dieters	Non-dieters
Weight Control Strategies at 11y	*n* = 44	*n* = 63	*n* = 30
Exercise*	22/44 (50.0)	4/63 (6.4)	0/30 (0)
Increase fruit/vegetable intake*	14/44 (31.8)	2/63 (3.2)	0/30 (0)
Eliminate snacking*	21/44 (47.7)	5/63 (7.9)	1/30 (3.3)
Eliminate sweets/junk*	22/44 (50.0)	3/63 (4.8)	1/30 (3.3)
Eat less food*	10/44 (22.7)	2/63 (3.2)	0/30 (0)
Eat low calorie foods*	7/44 (15.9)	3/63 (4.8)	0/30 (0)
Eat low fat foods*	14/44 (31.8)	1/63 (1.6)	0/30 (0)
Diet pills	0/44 (0)	0/63 (0)	0/30 (0)
Liquid diet*	5/44 (11.4)	0/63 (0)	0/30 (0)
Skip meals	4/44 (9.1)	1/63 (1.6)	0/30 (0)
Total strategies	2.70 (2.11)^A^	0.33 (1.11)^B^	0.07 (0.37)^B^
	*n* = 44	*n* = 64	*n* = 30
DEBQ at 11y			
Restraint	2.45 (0.65)^A^	1.62 (0.63)^B^	1.27 (0.31)^C^
Disinhibition	2.29 (0.52)^A^	1.91 (0.56)^B^	1.76 (0.50)^B^
chEAT at 11y			
Dieting	1.52 (2.03)^A^	0.17 (0.67)^B^	0.03 (0.18)^B^
Food Preoccupation	0.20 (0.41)	0.08 (0.41)	0.03 (0.18)
Social Pressure to Eat	0.34 (0.96)	0.31 (1.00)	0.07 (0.37)
Total	7.52 (1.81)^A^	4.58 (3.21)^B^	3.40 (1.81)^B^
Weight Concerns at 11y	1.24 (0.49)^A^	0.51 (0.41)^B^	0.29 (0.30)^C^
Weight Related Body Esteem 11y	2.61 (0.82)^A^	3.44 (0.50)^B^	3.64 (0.54)^B^

Values are mean (SD) or N (%). Means were compared using ANOVA and tukey’s post-hoc test. Values in the same row with different superscript letters are significantly different from one another (*p* < 0.05)

*Fisher’s exact test *p* < 0.05. Early dieters first dieted by age 11, adolescent dieters first dieted between 11 and 15 years

*DEBQ* Dutch Eating Behavior Questionnaire, *chEAT* Children’s Eating Behavior Questionnaire

### Anthropometric and physical activity predictors

Change in height and weight from 9 to 15 years, BMC at age 9 years, BMI percentile at age 15 years, and breast Tanner score at age 9 years were all significant predictors of ΔBMC from age 9 to 15 years (Table 3). A model including all of these parameters explained 63% of the variance in ΔBMC. Similarly, height at age 15 years, BMC at age 9 years, BMI percentile at age 15 years, and breast Tanner score at age 9 years were significant predictors of BMC at age 15 years, accounting for 79% of the variance (Table 4). Frequency of physical activity at age 9 did not significantly predict ΔBMC or BMC at 15.

**Table 3:** Association of dieting related variables with change in bone mineral content from 9 to 15 years

Model no.	Variable	B	95% CI	β	Semi-partial R^2^	*p*
1.	Base model (used in all analyses, R^2^ = 0.63)					
	Change in height from 9 to 15y (cm)	21.9	16.1, 27.7	0.55	0.10	< 0.0001
	Change in weight from 9 to 15y (kg)	3.77	0.55, 7.00	0.20	0.24	0.02
	BMC at 9y (g)	0.61	0.47, 0.75	0.53	0.26	< 0.0001
	BMI percentile at 15y	2.11	0.91, 3.30	0.31	0.03	0.0007
	Breast Tanner score at 9y	−26.9	−55.4, 1.5	− 0.13	0.003	0.06
	Frequency of physical activity at 9y	0.03	−2.31, 2.37	0.001	0.00	0.98
2.	Dieting variables (individually tested with base model)					
	Ever dieted	−25.5	−69.7, 18.8	−0.06	0.00	0.26
3.	Dieted by 11y	−46.9	−86.6, −7.1	− 0.13	0.01	0.02
4.	Dieted after 11y (*n* = 94, excludes early dieters)	−25.4	− 71.8, 21.0	− 0.07	0.00	0.28
5.	Number of weight control strategies used at 11y	−13.0	−23.7, − 2.21	−0.14	0.02	0.02
6.	DEBQ at 11y					
	Restraint	−35.0	−61.3, −8.81	−0.15	0.02	0.009
7.	Disinhibition	−24.7	−56.2, 6.88	−0.08	0.01	0.12
8.	chEAT at 11y					
	Dieting	−17.9	− 32.1, − 3.79	−0.15	0.02	0.01
9.	Food Preoccupation	−14.3	−63.8, 35.2	−0.03	0.00	0.57
10.	Social Pressure to Eat	−1.30	−21.9, 19.3	−0.01	0.00	0.90
11.	Total score	−3.17	−8.42, 2.08	−0.07	0.00	0.24
12.	Weight Concerns at 11y	−47.2	−82.9, −11.4	−0.16	0.02	0.01
13.	Weight-Related Body Esteem at 11y	23.5	−5.6, 52.6	0.11	0.01	0.10

Models 2–13 also include variables from model 1. *DEBQ* Dutch Eating Behavior Questionnaire, *chEAT* Children’s Eating Behavior Questionnaire

**Table 4:** Association of dieting related variables with bone mineral content at age 15 years

Model no.	Variable	B	95% CI	Std β	Semi-partial R^2^	*p*
1.	Base model (used in all analyses, R^2^ = 0.79)					
	Height at 15y (cm)	10.2	6.43, 13.9	0.26	0.22	< 0.0001
	BMC at 9y (g)	1.13	0.95, 1.31	0.64	0.44	< 0.0001
	BMI percentile at 15y	3.66	2.72, 4.61	0.35	0.09	< 0.0001
	Breast Tanner score at 9y	−76.4	−104.7, −48.0	−0.23	0.04	< 0.0001
	Frequency of physical activity at 9y	0.48	−2.19, 3.15	0.01	0.00	0.72
2.	Dieting variables (individually tested with base model)					
	Ever dieted	−40.8	−91.3, 9.70	−0.06	0.00	0.11
3.	Dieted by 11y	−65.4	−110.2, −20.7	−0.12	0.01	0.005
4.	Dieted after 11y (n = 94, excludes early dieters)	27.8	−79.2, 23.6	−0.05	0.00	0.29
5.	Number of weight control strategies used at 11y	−18.2	−30.4, −6.04	−0.13	0.01	0.004
6.	DEBQ at 11y					
	Restraint	−45.6	−75.2, −16.1	−0.13	0.01	0.003
7.	Disinhibition	−25.2	−61.3, 10.9	−0.06	0.00	0.17
8.	chEAT at 11y					
	Dieting	−22.5	−37.6, −7.34	−0.12	0.01	0.004
9.	Food Preoccupation	−53.1	−108.9, 2.70	−0.08	0.01	0.06
10.	Social Pressure to Eat	−0.09	−24.0, 23.8	−0.0003	0.00	0.99
11.	Total score	−5.89	−11.8, 0.01	−0.08	0.01	0.051
12.	Weight Concerns at 11y	−68.5	−108.4, −28.6	−0.15	0.02	0.0009
13.	Weight-Related Body Esteem at 11y	31.5	−1.03, 64.0	0.09	0.01	0.06

Models 2–13 also include variables from model 1. *DEBQ* Dutch Eating Behavior Questionnaire, *chEAT* Children’s Eating Behavior Questionnaire

### Dieting behaviors and BMC

Overall, girls who reported ever dieting by age 15 did not have significantly different ΔBMC than those that never dieted. However, there was a significant negative relationship between early dieting and ΔBMC (Table 3). After adjusting for anthropometric and physical activity variables, girls who were early dieters gained fewer grams of bone mineral from 9 to 15 years (M = 1071, 95% CI 1039–1103 g) than girls who were not early dieters (M = 1118, 95% CI 1096–1139 g, *p* = 0.02). The total number of weight control strategies girls had reported ever using at age 11 was also negatively associated with ΔBMC from age 9 to 15 years (B = − 13.0, 95% CI -23.7, − 2.21, p = 0.02). Greater restraint (B = − 35.0, 95% CI -61.3, − 8.81, *p* = 0.009), chEAT dieting score (B = − 17.9, 95% CI -32.1-3.79, p = 0.02), and weight concerns (B = − 47.2, 95% CI -82.9, − 11.4, *p* = 0.01) at age 11 years were all significantly associated with lower ΔBMC. Disinhibition, food preoccupation, social pressure to eat, and weight related body esteem were not associated with bone outcomes.

**Fig. 1:**
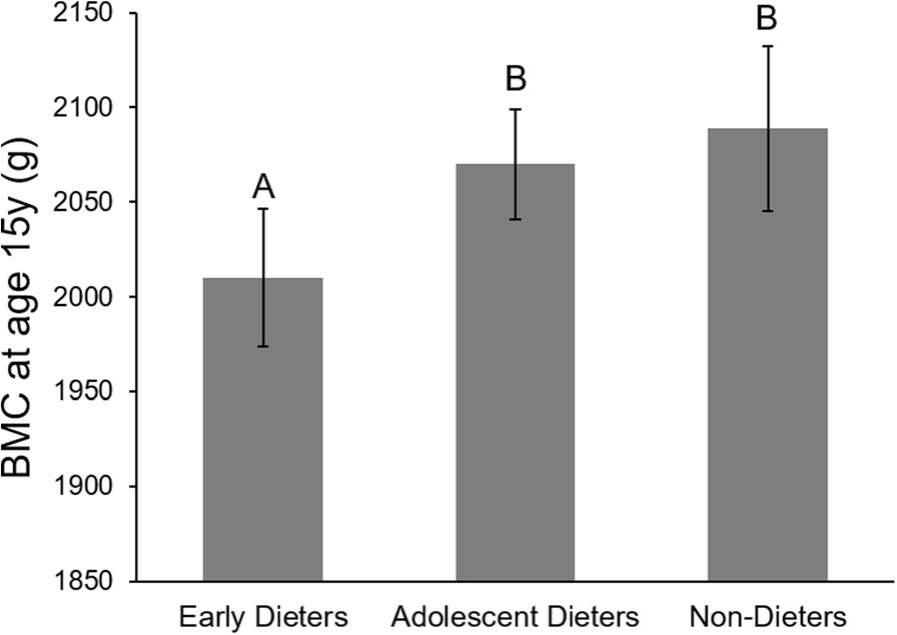
Girls who first dieted by age 11 (‘Early dieters’) have lower bone mineral content (BMC) at age 15 y than those who first dieted between 11 and 15 years (‘Adolescent dieters’) or not at all (‘Non-dieters’). Values are mean ± 95% confidence interval, adjusted for height and BMI percentile at age 15y, BMC at 9y, breast Tanner score at 9y, and habitual physical activity at 9 y

Similar patterns emerged using BMC at age 15 years as the outcome. Girls reported dieting at any age by 15 did not have significantly different BMC from those that never dieted, but early dieters had significantly lower BMC (M = 2008, 95% CI 1972–2044 g) than those that were not early dieters (M = 2073, 95% CI 2049–2098, *p* = 0.004, Fig. 1), a difference of 3.1%. As with ∆BMC, number of weight control strategies, restraint, chEAT dieting score and weight concerns at age 11 years were all negatively associated with BMC at 15 years (Table 4).

Because of the likely correlation between the various dieting measures, a stepwise model selection was performed to determine which dieting variables were the strongest predictors of bone outcomes. Results of the model selection found that only one dieting-related variable was retained in the model for each outcome (Table 5). For ΔBMC, the best-fitting model included restraint; for BMC at 15 years, the best-fitting model included weight concerns.

**Table 5:** Final models from stepwise regression

Variable	B	95% CI	Std β	Semi-partial R^2^	*p*
Outcome: Change in BMC 9–15 years (R^2^ = 0.64)					
Change in height from 9 to 15y (cm)	22.2	16.6, 27.7	0.56	0.11	< 0.0001
Change in weight from 9 to 15y (kg)	4.94	1.93, 7.96	0.26	0.23	0.002
BMC at 9y (g)	0.63	0.49, 0.77	0.55	0.25	< 0.0001
BMI percentile at 15y	1.93	0.81, 3.05	0.29	0.03	0.0009
Restraint at 11y	−37.0	−63.0, −10.9	−0.16	0.02	0.006
Outcome: BMC at 15 years (R^2^ = 0.81)					
Height at 15y (cm)	10.0	6.39, 13.7	0.25	0.23	< 0.0001
BMC at 9y (g)	1.21	1.02, 1.39	0.68	0.43	< 0.0001
BMI percentile at 15y	3.94	3.01, 4.86	0.38	0.09	< 0.0001
Breast Tanner score at 9y	−67.6	−95.6, −39.6	−0.21	0.04	< 0.0001
Weight concerns at 11y	−68.2	−108.0, −28.4	− 0.15	0.02	0.0009

### Dieting and bone-related nutrient and food group intake

As previously noted in this cohort [15], the majority of girls (86.3–89.9%) did not meet the Recommended Dietary Allowance of 1300 mg calcium at each of the assessment time points. Average calcium intake over age 9 to 15 years was 876 (SD 258) mg/day. Girls who reported dieting reported significantly lower (*p* < 0.05) average kcal, magnesium, and iron intakes than girls who never dieted, and there were also trends (*p* < 0.10) for lower intakes of calcium, potassium, zinc, fiber, and dairy among girls who dieted compared to non-dieters (Table 6). However, there were no differences in average nutrient intakes between early and adolescent dieters. Additionally, ΔBMC and BMC at age 15 years were not significantly predicted by reported intake of kilocalories, protein, bone-related micronutrients, or dairy intake (Additional file 1: Table S1 and Additional file 2: Table S2).

**Table 6:** Average nutrient intake by onset of dieting category

Average intake 9-15y	Non-dieters (*n* = 30)	Dieters			Non-dieters vs. all dieters, *p*
		All (*n* = 109)	Early (*n* = 44)	Adolescent (*n* = 65)	
Energy (kcal/day)	1819 (256)	1700 (255)	1730 (272)	1680 (243)	0.02
Protein (g/day)	62.9 (10.7)	60.9 (11.3)	62.7 (11.9)	59.6 (10.8)	0.39
Fiber (g/day)	12.8 (3.0)	11.7 (3.0)	11.6 (2.4)	11.7 (3.4)	0.07
Calcium (mg/day)	950 (305)	858 (242)	849 (232)	864 (250)	0.09
Vitamin D (μg/day)	5.1 (2.1)	4.8 (2.4)	4.9 (2.4)	4.7 (2.1)	0.49
Phosphorus (mg/day)	1104 (242)	1046 (225)	1050 (221)	1044 (230)	0.22
Magnesium (mg/day)	216 (51)	198 (41)	198 (37)	197 (44)	0.04
Sodium (mg/day)	2713 (499)	2663 (448)	2737 (432)	2613 (455)	0.59
Potassium (mg/day)	2088 (472)	1935 (427)	1970 (444)	1911 (417)	0.09
Vitamin C (mg/day)	74.1 (48.1)	62.9 (31.5)	59.0 (25.8)	65.5 (34.8)	0.13
Vitamin K (μg/day)	49.0 (21.0)	53.9 (25.7)	53.4 (20.7)	54.3 (28.7)	0.33
Iron (mg/day)	13.4 (3.0)	12.3 (2.6)	12.3 (2.2)	12.2 (2.8)	0.04
Zinc (mg/day)	9.6 (1.7)	8.9 (1.9)	9.0 (2.0)	8.8 (1.9)	0.05
Dairy (servings/day)	2.8 (1.0)	2.5 (0.8)	2.5 (0.8)	2.5 (0.9)	0.08

Values are mean (SD). Early dieters first dieted by age 11, adolescent dieters first dieted between 11 and 15 years. Differences in nutrient intake by dieting status were assessed by ANOVA. There were no significant differences between early and adolescent dieters

## Discussion

In this cohort of adolescent girls, early self-reported dieting was negatively related to bone mineral accrual from age 9 to 15 years and BMC at 15 years. Girls who reported dieting by age 11 years had 3% lower bone mineral content by age 15 than girls who did not diet in preadolescence. This percentage is similar in magnitude to the effects of calcium supplementation or physical activity interventions on bone mineral content in children and adolescents [3], and to the difference in total body BMC between girls with forearm fractures and age-matched controls [28], suggesting that this deficit in bone accrual associated with early dieting is potentially clinically significant. Additionally, we observed associations between several dieting-related behavioral characteristics in early adolescence and BMC. Greater dietary restraint, disordered eating attitudes, weight concerns, and number of weight control behaviors used, and lower weight-related body esteem were associated with lower BMC at age 15 years and lower bone mineral accrual across adolescence. In a stepwise model selection progress, dietary restraint and weight concerns were retained as the best dieting-related predictors of bone outcomes, likely because these continuous variables have more variability than the categorical self-reported dieting variable. Notably, restraint and weight concerns were the two variables at age 11 that differentiated all three dieting onset categories, with early dieters having the highest levels, non-dieters the lowest, and adolescent dieters having intermediate levels, despite not yet dieting at age 11. Though these characteristics may be stronger predictors of bone outcomes than dieting, self-reported dieting is derived from a single, yes/no question, which is easier to assess clinically than restraint or weight concerns, and may be more useful to screen children at risk for poorer bone acquisition.

To the best of our knowledge, this analysis is the first to look at longitudinal relationships between onset of dieting and bone health in adolescents. Previous cross-sectional studies have noted a relationship between a history of dieting and low bone mass or fractures in adults [10, 11]; however, none of these studies examined the influence of the timing of the onset of dieting. Our data suggest that girls who begin dieting in preadolescence are at elevated risk for impaired bone mineral accrual compared to girls who began dieting later in adolescence or did not diet in adolescence. This may be due to the emergence of dieting before peak calcium accretion, or to the influence of repeated dieting attempts; however, additional research is needed to confirm this.

Several studies have observed a relationship between dietary restraint and bone health in teenage [29] and adult [10, 29–31] women. In the current sample, dietary restraint was a significant negative predictor of bone mineral accrual in adolescents. In studies of young adult women, restraint has been associated with lower levels of bone formation markers [29, 32]. Thus, girls with higher dietary restraint may be at risk for lower bone mineral accrual during the period of peak bone growth, as well as for continued impairment of bone maintenance into adulthood. Additional research is needed to determine if girls who reported dieting before adolescence face additional risk of low bone mass if they continue to diet into adulthood.

Only a few studies have investigated the relationship between eating behavior measures and bone health in children. Barr et al. [33] studied changes in eating attitudes and bone mineral content in 45 white pre-menarcheal normal weight 9–12 year old girls over two years. They reported that, oral control, a subscale of the chEAT instrument that assesses self-control of food intake and social pressures to eat, was negatively associated with bone, predicting body size- and puberty-adjusted baseline, 2 year, and baseline-2 year change in total body and spinal BMC. In the current study, the relationship between this social pressure to eat and bone mineral accrual was not significant; however, the dieting subscale of chEAT was associated with smaller gains in BMC. We also found that weight concerns were negatively associated with bone mineral gains. This is similar to previous findings by Schvey et al. [34], who assessed cross-sectional relationships between disordered eating attitudes and lumbar bone mineral density in overweight African-American and Caucasian boys and girls ages 12–17. Shape concerns, but not restraint or other measures of eating attitudes, was a significant, negative predictor of lumbar BMD. The current findings that dieting and related eating behavior measures are predictive of bone mineral accrual in adolescent girls add to this body of evidence linking eating attitudes and bone health in adolescents.

There are a number of potential mechanisms through which dieting and related characteristics may impact bone acquisition. First, girls who diet may have lower intakes of nutrients required for adequate bone growth. In the present study, girls who reported dieting at any time between 9 and 15 years of age also reported lower intakes of several bone-related micronutrients than girls who did not diet, but there were no differences between early dieters and adolescent dieters in nutrient intakes, and nutrient intake was not a significant predictor of bone outcomes. Previous studies on the relationship between dieting and nutrient intake have had mixed findings. In adolescent girls, use of unhealthy weight control strategies (e.g. skipping meals, vomiting), but not healthy weight control strategies (e.g. exercise, increase fruits & vegetables), was associated with lower intakes of protein, calcium, iron, and zinc [35]. In another sample, adolescent girls who reported dieting had significantly higher intake of protein, but no differences in calcium or iron intake, compared to non-dieters, while adult female dieters had higher calcium intake than non-dieters [36]. The relationship between dieting and dietary intake is also complicated by underreporting, which may occur to a greater extent among dieters [37] and adolescents [38]. This has been evidenced in previous work with this cohort; underreporting was common in this sample and more prevalent in girls who use more weight control strategies [23] and girls with higher BMI [39, 40], dietary restraint, and weight concerns [40]. These data coupled with the fact that dietary intake of these nutrients did not predict ΔBMC or BMC at age 15 years suggest that 1) self-reported dietary intake does not accurately reflect nutrient status in this sample, and/or 2) reduced intake of bone-related nutrients is not the primary mechanism for reduced BMC among dieting adolescents.

Though we are unable to evaluate this hypothesis in the current data set, hormones may also provide a link between dieting and bone acquisition. In adult women, levels of the stress hormone cortisol have been positively associated with restraint [41, 42] and weight concerns [41]. Cortisol levels have been inversely associated with bone density in healthy children [43] and adult women [44]. Sex hormone abnormalities are associated with poorer bone health in anorexia nervosa patients [12], and have also been proposed as a mechanism between dietary restraint and bone health in adult women [31, 42]. However, we observed relationships between dieting/dietary restraint measured at age 11, when the majority of our participants were in early puberty, and bone acquisition through age 15. Additionally, earlier pubertal development has been associated with increased risk of dieting in early adolescence [45], but also *greater* bone mineral accrual [46]. Future studies are needed to determine if the relationship between dieting in early adolescence and bone mineral accrual is mediated by hormones.

There are limitations to this study. The sample was relatively small and primarily non-Hispanic white, two-parent families with middle to high incomes, which limits the generalizability of these findings to other groups. Though the data used for this analysis were collected from 2000 to 2007, the conclusions remain relevant today given the increasing prevalence of weight loss attempts [8] and continued inadequate intake of bone-related nutrients [47] among adolescents. Additionally, dieting was self-reported by participants, and thus the true nature and extent of their dieting behavior is unknown. However, the present analysis as well as previous findings linking cognitive restraint to bone health suggest that cognitions about dieting may be just as important as dieting behavior itself.

## Conclusions

In summary, dieting in preadolescence is associated smaller gains in BMC across adolescence and lower BMC at age 15 years. This study builds on previous findings that measures of disordered eating attitudes in healthy children are associated with poorer bone health. Further research is needed to elucidate mechanisms behind this observation. Interventions to prevent dieting in preadolescents and adolescents may also improve bone health. In light of the ongoing childhood obesity crisis, additional research is needed to identify weight management strategies that do not interfere with bone health in children.

## Additional files

Additional file 1: **Table S1.** Dietary intake predictors of change in bone mineral content from 9 to 15 years. Results of regression analyses predicting change in bone mineral content from 9 to 15 years from dietary intake of bone-related nutrients/food groups, adjusting for anthropometric, pubertal development, and physical activity variables, in girls. (DOCX 13 kb)

Additional file 2: **Table S2.** Dietary intake predictors of bone mineral content at age 15 years. Results of regression analyses predicting bone mineral content at age 15 years from dietary intake of bone-related nutrients/food groups, adjusting for anthropometric, pubertal development, and physical activity variables, in girls. (DOCX 13 kb)

## Abbreviations

BMC: Bone mineral content
BMD: Bone mineral density
BMI: Body mass index
chEAT: Children’s Eating Attitude Test
DEBQ: Dutch Eating Behavior Questionnaire
DXA: Dual-energy x-ray absorptiometry
