# Supplementary material for: Dieting is associated with reduced bone mineral accrual in a longitudinal cohort of girls
Source: BMC Public Health. 2018 Nov 22;18:1285. doi: 10.1186/s12889-018-6206-y (PMC6251190; doi:10.1186/s12889-018-6206-y)
Supplement: Supplementary file 1 — Table S1. Dietary intake predictors of change in bone mineral content from 9 to 15 years. Results of regression analyses predicting change in bone mineral content from 9 to 15 years from dietary intake of bone-related nutrients/food groups, adjusting for anthropometric, pubertal development, and physical activity variables, in girls. (DOCX 13 kb) [file 12889_2018_6206_MOESM1_ESM.docx]

Additional file 1

Table S1 – Dietary intake predictors of change in bone mineral content from 9 to 15 years

| Model no. | Variable | B | 95% CI | Std β | Semi-partial R^2^ | p |
| --- | --- | --- | --- | --- | --- | --- |
| 1. | **Base model (used in all analyses, R^2^=0.63)** | | | | | |
|  | Change in height from 9 to 15y (cm) | 21.9 | 16.1, 27.7 | 0.55 | 0.10 | **<0.0001** |
|  | Change in weight from 9 to 15y (kg) | 3.77 | 0.55, 7.00 | 0.20 | 0.24 | **0.02** |
|  | BMC at 9y (g) | 0.61 | 0.47, 0.75 | 0.53 | 0.26 | **<0.0001** |
|  | BMI percentile at 15y | 2.11 | 0.91, 3.30 | 0.31 | 0.03 | **0.0007** |
|  | Breast Tanner score at 9y | -26.9 | -55.4, 1.5 | -0.13 | 0.003 | 0.06 |
|  | Frequency of physical activity at 9y | 0.03 | -2.31, 2.37 | 0.001 | 0.00 | 0.98 |
|  | **Dietary intake variables (individually tested with base model)** | | | | | |
| 2. | Energy (kcal/day) | 0.05 | -0.02, 0.13 | 0.07 | 0.005 | 0.19 |
| 3. | Protein (g/day) | 1.19 | -0.43, 2.81 | 0.08 | 0.006 | 0.15 |
| 4. | Fiber (g/day) | -1.39 | -7.39, 4.61 | -0.03 | 0.0006 | 0.65 |
| 5. | Calcium (mg/day) | 0.03 | -0.04, 0.11 | 0.05 | 0.003 | 0.33 |
| 6. | Vitamin D (µg/day) | 1.95 | -6.33, 10.2 | 0.03 | 0.006 | 0.64 |
| 7. | Phosphorus (mg/day) | 0.03 | -0.06, 0.11 | 0.03 | 0.001 | 0.54 |
| 8. | Magnesium (mg/day) | 0.02 | -0.40, 0.44 | 0.004 | 0.00002 | 0.94 |
| 9. | Sodium (mg/day) | 0.007 | -0.03, 0.05 | 0.02 | 0.0003 | 0.73 |
| 10. | Potassium (mg/day) | 0.008 | -0.03, 0.05 | 0.02 | 0.0004 | 0.69 |
| 11. | Vitamin C (mg/day) | 0.29 | -0.20, 0.79 | 0.06 | 0.004 | 0.25 |
| 12. | Vitamin K (µg/day) | 0.41 | -0.33, 1.15 | 0.06 | 0.003 | 0.28 |
| 13. | Iron (mg/day) | -0.37 | -7.08, 6.33 | -0.006 | 0.00003 | 0.91 |
| 14. | Zinc (mg/day) | 3.64 | -6.19, 13.5 | 0.04 | 0.002 | 0.46 |
| 15. | Dairy (servings/day) | 7.01 | -13.5, 27.5 | 0.04 | 0.001 | 0.50 |

Models 2-15 also include all variables from model 1.
