# Supplementary material for: Dieting is associated with reduced bone mineral accrual in a longitudinal cohort of girls
Source: BMC Public Health. 2018 Nov 22;18:1285. doi: 10.1186/s12889-018-6206-y (PMC6251190; doi:10.1186/s12889-018-6206-y)
Supplement: Supplementary file 2 — Table S2. Dietary intake predictors of bone mineral content at age 15 years. Results of regression analyses predicting bone mineral content at age 15 years from dietary intake of bone-related nutrients/food groups, adjusting for anthropometric, pubertal development, and physical activity variables, in girls. (DOCX 13 kb) [file 12889_2018_6206_MOESM2_ESM.docx]

Additional file 2: Table S2 – Dietary intake predictors of bone mineral content at age 15 years

| Model no. | Variable | B | 95% CI | Std β | Semi-partial R^2^ | p |
| --- | --- | --- | --- | --- | --- | --- |
| 1. | **Base model (used in all analyses, R^2^=0.79)** | | | |  |  |
|  | Height at 15y (cm) | 10.2 | 6.43, 13.9 | 0.26 | 0.22 | **<0.0001** |
|  | BMC at 9y (g) | 1.13 | 0.95, 1.31 | 0.64 | 0.44 | **<0.0001** |
|  | BMI percentile at 15y | 3.66 | 2.72, 4.61 | 0.35 | 0.09 | **<0.0001** |
|  | Breast Tanner score at 9y | -76.4 | -104.7, -48.0 | -0.23 | 0.04 | **<0.0001** |
|  | Frequency of physical activity at 9y | 0.48 | -2.19, 3.15 | 0.01 | 0.00 | 0.72 |
|  | **Dietary intake variables (individually tested with base model)** | | | | | |
| 2. | Energy (kcal/day) | 0.03 | -0.05, 0.12 | 0.03 | 0.001 | 0.42 |
| 3. | Protein (g/day) | 1.71 | -0.14, 3.56 | 0.07 | 0.005 | 0.07 |
| 4. | Fiber (g/day) | -1.78 | -8.72, 5.16 | -0.02 | 0.0004 | 0.61 |
| 5. | Calcium (mg/day) | 0.06 | -0.02, 0.14 | 0.06 | 0.003 | 0.14 |
| 6. | Vitamin D (µg/day) | 6.01 | -3.39, 15.4 | 0.05 | 0.002 | 0.21 |
| 7. | Phosphorus (mg/day) | 0.05 | -0.04, 0.14 | 0.04 | 0.002 | 0.28 |
| 8. | Magnesium (mg/day) | 0.13 | -0.35, 0.62 | 0.02 | 0.0005 | 0.59 |
| 9. | Sodium (mg/day) | 0.0003 | -0.05, 0.05 | 0.0005 | 2x10^-7^ | 0.99 |
| 10. | Potassium (mg/day) | 0.02 | -0.03, 0.07 | 0.03 | 0.001 | 0.44 |
| 11. | Vitamin C (mg/day) | 0.25 | -0.32, 0.83 | 0.03 | 0.001 | 0.38 |
| 12. | Vitamin K (µg/day) | 0.33 | -0.54, 1.20 | 0.03 | 0.001 | 0.45 |
| 13. | Iron (mg/day) | 1.48 | -6.22, 9.19 | 0.02 | 0.0002 | 0.70 |
| 14. | Zinc (mg/day) | 8.70 | -2.40, 19.8 | 0.06 | 0.004 | 0.12 |
| 15. | Dairy (servings/day) | 13.0 | -10.5, 39.4 | 0.04 | 0.002 | 0.28 |

Models 2-15 also include all variables from model 1.
